# Explaining ethnic variations in adolescent mental health: a secondary analysis of the Millennium Cohort Study

**DOI:** 10.1007/s00127-021-02167-w

**Published:** 2021-10-24

**Authors:** Gargie Ahmad, Sally McManus, Laia Bécares, Stephani L. Hatch, Jayati Das-Munshi

**Affiliations:** 1 Violence and Society Centre, City, University of London, and National Centre for Social Research, London, UK; 2 Department of Social Work and Social Care, University of Sussex, Brighton, UK; 3 Department of Psychological Medicine, Institute of Psychiatry, Psychology and Neuroscience, King’s College London, 16 De Crespigny Park, London, SE5 8AF UK; 4 ESRC Centre for Society and Mental Health, King’s College London, London, UK

**Keywords:** Ethnicity, Mental health, Adolescence, Social factors, Inequalities

## Abstract

**Purpose:**

The relationship between ethnicity and adolescent mental health was investigated using cross-sectional data from the nationally representative UK Millennium Cohort Study.

**Methods:**

Parental Strengths and Difficulties Questionnaire reports identified mental health problems in 10,357 young people aged 14 (*n* = 2042 from ethnic minority backgrounds: Mixed *n* = 492, Indian *n* = 275, Pakistani *n* = 496, Bangladeshi *n* = 221, Black Caribbean *n* = 102, Black African *n* = 187, Other Ethnic Group *n* = 269). Univariable logistic regression models investigated associations between each factor and outcome; a bivariable model investigated whether household income explained differences by ethnicity, and a multivariable model additionally adjusted for factors of social support (self-assessed support, parental relationship), participation (socialising, organised activities, religious attendance), and adversity (bullying, victimisation, substance use). Results were stratified by sex as evidence of a sex/ethnicity interaction was found (*P* = 0.0002).

**Results:**

There were lower unadjusted odds for mental health problems in boys from Black African (OR 0.15, 95% CI 0.04–0.61) and Indian backgrounds (OR 0.42, 95% CI 0.21–0.86) compared to White peers. After adjustment for income, odds were lower in boys from Black African (OR 0.10, 95% CI 0.02–0.38), Indian (OR 0.40, 95% CI 0.21–0.77), and Pakistani (OR 0.49, 95% CI 0.27–0.89) backgrounds, and girls from Bangladeshi (OR 0.18, 95% CI 0.05–0.65) and Pakistani (OR 0.63, 95% CI 0.41–0.99) backgrounds. After further adjustment for social support, participation, and adversity factors, only boys from a Black African background had lower odds (OR 0.16, 95% CI 0.03–0.71) of mental health problems.

**Conclusions:**

Household income confounded lower prevalence of mental health problems in some young people from Pakistani and Bangladeshi backgrounds; findings suggest ethnic differences are partly but not fully accounted for by income, social support, participation, and adversity. Addressing income inequalities and socially focused interventions may protect against mental health problems irrespective of ethnicity.

**Supplementary Information:**

The online version contains supplementary material available at 10.1007/s00127-021-02167-w.

## Introduction

As mental health problems such as anxiety and depression often first manifest by adolescence, an improved understanding is required to address inequalities and achieve lifelong beneficial effects for young people [1–4]. In England, the prevalence of mental disorder is higher in children aged 5–19 from disadvantaged socioeconomic backgrounds, who are also more likely to experience problems with family functioning, adverse life events, and reduced social support and participation. However, the overall prevalence of mental health problems is lower for children from Black and Asian backgrounds compared to their White British peers [5].

A large body of evidence has explored associations between strong social relationships and better mental health, and experience of social adversity and socioeconomic disadvantage with worse mental health [6]. The relationship between ethnicity and mental health remains more complex. In the UK, people from ethnic minority backgrounds are more likely to face socioeconomic adversity than their White British peers, and are more likely to be living in deprived areas [7]. Ethnic minority status exposes young people to experiences of racism, discrimination, and social marginalisation, which have been associated with adverse mental health problems [8, 9]. The lower prevalence of mental health problems in young people from some ethnic minority groups therefore run counter to expectation.

The last systematic review of ethnic inequalities in child and adolescent mental health found that children from Black African and Indian backgrounds had better mental health, while children from Mixed, Black Caribbean, Pakistani and Bangladeshi backgrounds had similar levels of mental health problems, compared to their White British peers; overall no disadvantage in mental health problems was found in these groups, despite the overall increased adversity that might otherwise predispose them to developing problems [10].

Evidence has remained mixed in the decade since: in east London, young people from Bangladeshi and Indian backgrounds had a reduced likelihood of reporting psychological distress and mental health problems, whereas those from White Other backgrounds had a higher likelihood, compared to their White British peers [11, 12]. Investigators in another London wide study observed a lower prevalence of mental health problems in boys from Black Caribbean, Nigerian, and Ghanaian backgrounds, and girls from Indian backgrounds [13].

Findings of lower prevalence of mental health problems in some ethnic minority adolescents are persistent across studies using different instruments to measure mental health. However, data from highly diverse, multi-cultural urban contexts such as London have largely been used in investigations to date, which may not have captured national differences. To address this, data were analysed from the Millennium Cohort Study (MCS), a multi-purpose longitudinal study following a large, nationally representative sample of young people. This is the first analysis exploring the potential explanatory mechanisms of social support, participation, and adversity behind the relationship between ethnicity and mental health, by investigating young people’s self-reported social activities, relationships, and lifestyle.

We hypothesised that there would be lower prevalence of mental health problems in young people from some ethnic minority backgrounds compared to White peers, and that these associations would be attenuated by adjustment for factors of social support, participation, adversity, and household income.

## Methods

### Data source

The MCS is a nationally representative longitudinal birth cohort study, following children born across the UK between 2000 and 2002. Using the child benefit register as sampling frame, MCS covered most of the child population except those not registered due to recent, temporary, or irregular immigration status [14]. A stratified, clustered, random sampling strategy, purposefully oversampling economically disadvantaged and ethnically diverse areas (where ethnic minority representation was over 30% in the 1991 census) was used. Further information is provided in survey documentation from the Centre for Longitudinal Studies (CLS) [15].

From an initial 19,519 participating families, responses in the sixth sweep were provided by 11,884 families, which collected information on cohort members aged around 14 years. Data collection was conducted between January 2015 and April 2016. This analysis used data from parent responses to the Household and Parental Questionnaire, and self-completed responses by cohort members to the Young Person Questionnaire.

### Measures

Mental health problems were measured through parent responses to the Strengths and Difficulties Questionnaire (SDQ), a validated psychometric tool providing a dimensional measure of mental health in children aged 3–16 [16]. Scores are measured for 25 items over conduct, hyperactivity/inattention, emotional, peer, and pro-social problems subscales. Scores across all except the pro-social subscale are added to obtain a Total Difficulties Score (TDS), between 0 and 40; TDS of 17 or above is indicative of probable clinically diagnosable mental disorder [17]. The SDQ is widely used in child and adolescent mental health research, and other studies investigating ethnic mental health inequalities [18].

Eight harmonised categories derived by MCS, based on Office for National Statistics categories used by young people to self-report ethnicity, were used to describe ethnic background: White (including White British, Irish, Gypsy or Irish Traveller, and White Other), Mixed (including any multiple ethnic background), Black African, Black Caribbean, Indian, Pakistani, Bangladeshi, and Other Ethnic Group (including Arab, Chinese, Other Black, or Other Asian).

Household income was measured using Organisation for Economic Co-operation and Development (OECD) equivalised income quintiles, which adjusts weekly household income data according to size, composition, and resource needs. Similar equivalisation indicates comparable standards of living [19].

### Social support and participation factors

To measure social support, cohort members were asked three question items from the Social Provisions Scale, detecting presence and absence of social support: ‘*I have family and friends that help me feel safe, secure, and happy*’; ‘*There is someone I trust whom I would turn to for advice if I were having problems*’; ‘*There is no one I feel close to*’ [20]. Responses ranged from ‘*Very true*’, ‘*Partly true*’, and ‘*Not true at all*’. Scores were summed for questions detecting presence and reversed prior to summing for absence. Higher scores correlate to a greater degree of social support. Total scores of 8–9 were used to indicate strong social support, 6–7 for some social support, and 5 or less as indicative of little to no social support.

To assess parental relationship, responses to the question *‘Overall, how close would you say you are to your mother/father?’* were grouped as: *‘Not close to either parent/no parents’, ‘Close to one parent’, or ‘Close to both parents’.*

Social participation was measured through amount of time cohort members spent with friends outside school, in organised activities such as youth clubs, scouts, girl guides or other activities, and attendance of a religious service.

### Social adversity factors

Bullying was measured as the current frequency of being picked on or hurt by other children, received unwanted or nasty online or social media communications, and a separate bullying measure for having been the perpetrators of these actions.

Victimisation was measured through the cumulative number of experiences of being insulted, called names, threatened, or shouted at in a public place, school, or elsewhere, physical violence, having something stolen, unwanted sexual approaches or sexual assault in the past year.

Substance use was measured as number of experiences of smoking of 1–6 cigarettes a week or more, drinking 5 or more alcoholic drinks at one time, trying cannabis more than 5 times, and trying any other illegal drugs in the past year.

### Statistical analyses

Analysis was conducted in Stata 14. Survey weights were applied to address sample design, and bias related to sampling and attrition [15]. Further weighting information can be found in survey documentation [21].

Distribution of all measures by ethnicity was described. Pearson Chi-squared tests initially assessed any differences here, and for crude associations between each measure and having mental health problems (see Supplementary table 1). Univariable logistic regressions were then used to investigate unadjusted measures of effect of ethnicity, sex, income, and all social factors with mental health problems respectively.

In the adjusted analysis, a bivariable logistic regression model was used to explore the relationship between ethnicity and mental health problems adjusted for household income only, and a multivariable model additionally adjusted for income and all social factors (originally, models separately tested social support and participation factors and social adversity factors, which were both, respectively, significant, so were included altogether here).

**Fig. 1:**
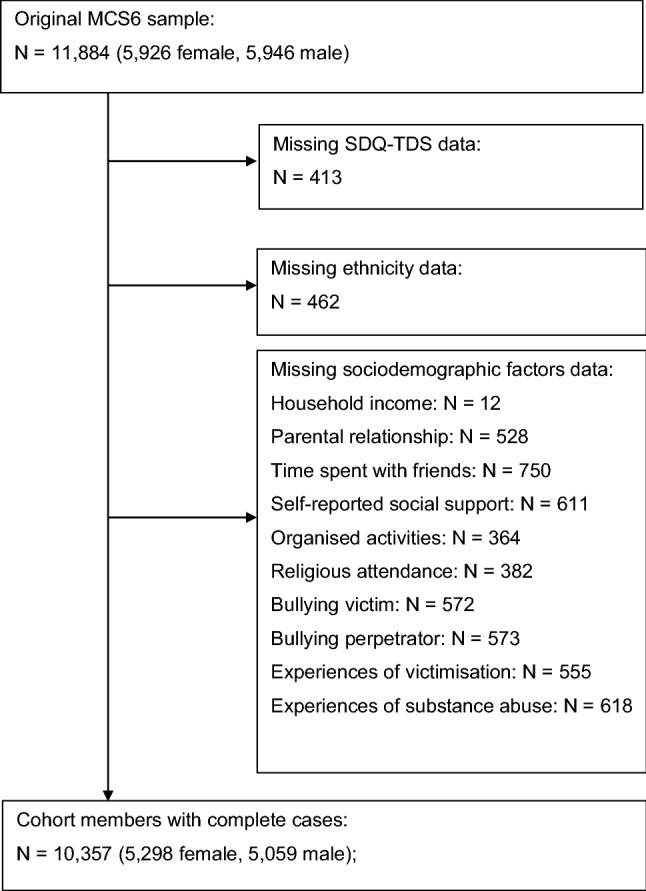
Sample flow diagram, complete case MCS6 sample for explaining ethnic variations in adolescent mental health: a secondary analysis of the Millennium Cohort Study

Wald tests were applied to assess overall associations of each factor with the outcome. We assessed interactions between ethnicity and sex across all models and found evidence in support of effect modification. Sex stratified results are therefore presented across all adjusted models. Complete case analysis of data from 10,357 cohort members (87.2% of the overall cohort), who provided complete information across all measures, was conducted (Fig. 1).

Finally, we conducted a series of supplementary analyses assessing count of symptoms using Poisson regression to investigate the SDQ total score, externalising and internalising subscales as linear measures of mental health outcomes as a sensitivity analysis.

## Results

Of the total sample of 10,357 young people, *n* = 5298 (49.6%) were female and *n* = 5059 (50.4%) were male (survey weighted proportions are detailed here, see Table 1). 2,042 were from ethnic minority backgrounds: *n* = 492 (5.4%) Mixed, *n* = 275 (2.1%) Indian, *n* = 496 (3.3%) Pakistani, *n* = 221 (1.4%) Bangladeshi, *n* = 102 (1.3%) Black Caribbean, *n* = 187 (2.3%) Black African, and *n* = 269 (2.8%) Other Ethnic Group backgrounds, respectively; *n* = 8315 (81.5%) were from White backgrounds, considered the reference group.

**Table 1:** Distribution of demographic and social factors by ethnicity in MCS6 (sample *n* = 10,357, weighted column proportions, %)

Ethnicity	White (*n* = 8315, 81.5%)	Mixed (*n* = 492, 5.4%)	Indian (*n* = 275, 2.1%)	Pakistani (*n* = 496, 3.3%)	Bangladeshi (*n* = 221, 1.4%)	Black Caribbean (*n* = 102, 1.3%)	Black African (*n* = 187, 2.3%)	Other Ethnic Group (*n* = 269, 2.8%)	Total sample (*N* = 10,357)	Chi-squared adjusted design-based F
Factor										
Prevalence of mental health problems (SDQ TDS ≥ 17)										
*n*	665	49	16	55	15	13	5	31	849	3.4 (*P* = 0.0040)
%	10.0	11.0	4.5	10.2	8.0	15.8	2.9	14.0	9.9	
(95% CI)	(9.0–11.0)	(8.0–14.6)	(2.5–7.8)	(8.1–12.9)	(5.9–10.7)	(8.8–26.9)	(1.1–7.3)	(9.0–21.3)	(9.1–10.6)	
Distribution of demographic and social factors by ethnicity										
Sex										
Female	4257 (50.0)	246 (45.1)	130 (44.2)	265 (53.4)	120 (52.2)	47 (42.2)	93 (46.8)	140 (49.4)	5298 (49.6)	
Male	4058 (50.0)	246 (54.9)	145 (55.8)	231 (46.6)	101 (47.8)	55 (57.8)	94 (53.2)	129 (50.7)	5059 (50.4)	1.2 (*P* = 0.33)
Household income										
Lowest quintile	821 (13.1)	94 (20.4)	20 (9.1)	324 (64.2)	145 (70.2)	31 (40.2)	73 (47.4)	75 (31.9)	1583 (17.5)	
Second quintile	1262 (18.8)	95 (20.8)	60 (26.8)	122 (23.9)	52 (20.8)	23 (22.8)	37 (20.9)	52 (21.1)	1703 (19.4)	
Third quintile	1792 (21.3)	99 (20.9)	64 (20.8)	37 (9.8)	17 (5.2)	23 (20.4)	35 (14.2)	60 (20.0)	2127 (20.5)	
Fourth quintile	2219 (23.2)	94 (17.6)	67 (23.6)	11 (2.0)	5 (2.5)	18 (13.1)	25 (9.4)	50 (17.6)	2489 (21.3)	
Highest quintile	2221 (23.6)	110 (20.4)	64 (19.7)	2 (0.1)	2 (1.3)	7 (3.7)	17 (8.2)	32 (9.5)	2455 (21.3)	23.3 (*P* < 0.0001)
Parental relationship										
Not very close to either/no parents	115 (1.8)	14 (3.4)	5 (2.0)	11 (2.0)	4 (1.8)	4 (5.8)	5 (3.2)	4 (1.4)	162 (1.9)	
Close to one parent	1301 (19.1)	111 (25.2)	32 (13.0)	81 (15.8)	25 (10.6)	32 (39.4)	36 (22.7)	44 (22.2)	1662 (19.5)	
Close to both parents	6899 (79.1)	367 (71.3)	238 (85.1)	404 (82.2)	192 (87.6)	66 (54.8)	146 (74.2)	221 (76.4)	8533 (78.5)	3.5 (*P* = 0.0002)
Social support										
Little/no social support	83 (1.1)	11 (1.4)	4 (0.9)	9 (1.6)	2 (0.8)	2 (3.7)	1 (0.8)	5 (2.4)	117 (1.2)	
Some social support	882 (11.9)	69 (13.5)	29 (12.4)	58 (11.6)	24 (9.8)	18 (19.5)	21 (14.6)	46 (15.6)	1147 (12.2)	
Strong social support	7350 (87.0)	412 (85.1)	242 (86.6)	429 (86.9)	195 (89.4)	82 (76.8)	165 (84.5)	218 (81.9)	9093 (86.6)	1.2 (*P* = 0.31)
Time spent with friends outside school										
Never	171 (2.3)	22 (3.5)	16 (4.8)	72 (14.3)	28 (13.3)	3 (2.8)	6 (3.7)	23 (7.5)	341 (3.1)	
Monthly	1862 (21.3)	114 (21.1)	118 (38.6)	163 (30.2)	87 (33.8)	28 (29.9)	66 (31.6)	89 (31.5)	2527 (22.8)	
Weekly or more	6282 (76.4)	356 (75.3)	141 (56.6)	261 (55.5)	106 (52.9)	71 (67.3)	115 (64.7)	157 (61.0)	7489 (74.1)	14.3 (*P* < 0.0001)
Time spent in organised activities										
Never/almost never	3498 (46.0)	207 (44.4)	117 (42.0)	254 (53.8)	98 (40.7)	29 (24.8)	66 (41.4)	101 (37.4)	4370 (45.4)	
Between once a year-once a month	1485 (17.9)	99 (18.8)	76 (26.0)	139 (25.3)	73 (30.1)	29 (37.9)	57 (26.7)	69 (26.4)	2027 (19.3)	
At least weekly	3332 (36.1)	186 (36.8)	82 (32.0)	103 (20.9)	50 (29.2)	44 (37.3)	64 (31.9)	99 (36.3)	3960 (35.4)	4.7 (*P* < 0.0001)
Time spent in religious attendance										
Never/almost never	5063 (67.0)	240 (54.5)	41 (17.3)	45 (10.0)	34 (14.5)	34 (41.1)	14 (8.9)	86 (36.9)	5557 (60.2)	
Between once a year-once a month	2174 (26.1)	164 (30.8)	152 (52.7)	183 (34.3)	82 (34.6)	44 (35.7)	41 (23.9)	100 (29.3)	3163 (27.5)	
At least weekly	855 (6.9)	88 (14.8)	82 (30.0)	268 (55.7)	105 (50.9)	24 (23.2)	132 (67.2)	83 (33.8)	1637 (12.4)	80.1 (*P* < 0.0001)
Being a victim of bullying										
Never	3439 (41.4)	223 (46.9)	152 (60.2)	298 (61.6)	129 (59.0)	57 (60.7)	112 (60.1)	139 (53.0)	4549 (44.0)	
Less often	2820 (33.5)	152 (31.5)	88 (26.5)	126 (23.7)	70 (31.7)	27 (19.9)	48 (24.6)	82 (31.7)	3413 (32.5)	
Every few months	617 (7.1)	34 (6.2)	12 (4.8)	18 (3.0)	8 (3.1)	8 (7.8)	8 (5.6)	16 (4.5)	721 (6.7)	
About once a month	526 (6.4)	29 (5.4)	8 (2.5)	13 (3.0)	5 (1.6)	5 (7.5)	7 (2.2)	9 (3.1)	602 (5.9)	
About once a week	550 (6.6)	31 (5.2)	10 (3.1)	20 (4.5)	4 (2.6)	4 (3.6)	8 (4.3)	15 (5.6)	642 (6.2)	
Most days	363 (5.1)	23 (4.8)	5 (2.8)	21 (4.2)	5 (2.0)	1 (0.5)	4 (3.3)	8 (2.1)	430 (4.7)	3.5 (*P* < 0.0001)
Being a perpetrator of bullying										
Never	5528 (65.3)	302 (60.0)	188 (66.6)	338 (70.5)	151 (70.8)	64 (62.7)	129 (63.7)	168 (59.3)	6868 (65.1)	
Less often	2092 (26.0)	120 (25.7)	67 (24.7)	119 (22.0)	56 (22.5)	25 (21.6)	40 (24.6)	76 (30.2)	2595 (25.8)	
Every few months	273 (3.2)	22 (5.1)	7 (3.3)	11 (1.9)	4 (1.2)	6 (4.8)	6 (5.0)	8 (2.8)	337 (3.3)	
About once a month	185 (2.4)	21 (3.5)	5 (1.3)	9 (2.2)	3 (1.4)	4 (9.0)	6 (1.6)	7 (3.4)	240 (2.5)	
About once a week	166 (2.1)	17 (3.4)	6 (2.5)	12 (2.0)	6 (3.7)	2 (1.4)	5 (4.3)	5 (1.9)	219 (2.2)	
Most days	71 (1.0)	10 (2.2)	2 (1.7)	7 (1.5)	1 (0.4)	1 (0.5)	1 (0.8)	5 (2.4)	98 (1.1)	1.5 (*P* = 0.074)
Experiences of victimisation										
None	4102 (48.6)	238 (44.2)	177 (66.6)	337 (70.0)	155 (74.2)	52 (55.9)	101 (57.7)	170 (59.3)	5332 (50.4)	
One	2391 (28.5)	133 (30.6)	60 (20.6)	90 (18.0)	44 (16.8)	26 (18.7)	51 (24.5)	55 (21.7)	2850 (27.5)	
Two	1305 (15.7)	80 (18.1)	29 (9.6)	54 (9.8)	19 (6.9)	19 (22.0)	24 (11.9)	26 (12.5)	1556 (15.3)	
Three or more	517 (7.2)	41 (7.2)	9 (3.3)	15 (2.3)	3 (2.1)	5 (3.4)	11 (5.9)	18 (6.6)	619 (6.8)	5.1 (*P* < 0.0001)
Experiences of substance use										
None	7261 (85.7)	423 (85.7)	270 (96.9)	485 (97.7)	217 (98.6)	89 (84.9)	176 (94.9)	248 (91.5)	9169 (86.9)	
One	742 (9.6)	41 (8.8)	1 (0.1)	8 (1.5)	4 (1.4)	8 (7.2)	10 (4.3)	10 (3.9)	824 (8.6)	
Two	216 (3.1)	20 (4.3)	2 (1.3)	2 (0.7)	0 (0.0)	5 (7.9)	1 (0.8)	7 (3.1)	253 (3.0)	2.7 (*P* = .0035)
Three or more	96 (1.6)	8 (1.2)	2 (1.7)	1 (0.2)	0 (0	0 (0.0)	0 (0.0)	4 (1.6)	111 (1.5)	

Social factors largely varied by ethnicity, as detailed in Table 1. Remarkably high proportions of young people from Bangladeshi (70.2%) and Pakistani (64.2%) backgrounds were in the lowest household income quintile, and higher proportions of those from Black African (47.4%), Black Caribbean (40.2%), and Other Ethnic Group (31.9%) backgrounds were also in this category, compared with White peers (13.1%). Young people from a Bangladeshi background were the largest proportion (87.6%), and those from a Black Caribbean background the smallest proportion (54.8%), reporting being close to both parents (79.1% in White peers). Overall, while findings were mixed for social support and organised activities, except for those from Mixed backgrounds, smaller proportions of all young people from other ethnic minority backgrounds reported spending time weekly with friends outside school. Larger proportions of those from all ethnic minority backgrounds spent time weekly in religious attendance. Larger proportions of those from ethnic minority backgrounds (except for those from Mixed backgrounds) reported they had never been the victim of bullying (findings were mixed on being a perpetrator) and had no experiences of victimisation or substance use (see Supplementary Tables 2, 3 for breakdown of social factors by sex).

Differences in prevalence of mental health problems according to each social factor are provided in the supplementary material (Supplementary Table 1). Prevalence of mental health problems was 9.9% (95% confidence intervals (CI) 9.1–10.6%) for all cohort members, which was lower in girls at 9.0% (95% CI 8.0–10.0%) and higher in boys at 10.9% (95% CI 9.6–12.2%). Prevalence varied by ethnicity and was indicatively highest in young people from Black Caribbean backgrounds at 15.8% (95% CI 8.8–26.9%) and lowest in those from Black African backgrounds at 2.9% (95% CI 1.1–7.3%).

In the unadjusted logistic regression analyses of outcome and ethnicity, there were significantly lower odds of mental health problems for Black African (odds ratio (OR) 0.15, 95% Cl 0.04–0.61) and Indian (OR 0.42, 95% CI 0.21–0.86) boys, compared to their White peers (model 1, Table 2). Lower odds were also found in boys from Mixed and Pakistani backgrounds, and girls from Bangladeshi, Black African, and Indian backgrounds; higher odds were found in boys from Bangladeshi, Black Caribbean, and Other Ethnic backgrounds, and girls from Mixed, Pakistani, Black Caribbean, and Other Ethnic backgrounds, however the 95% confidence intervals for these OR estimates were wide.

**Table 2:** Logistic regression results summary of association between ethnicity and mental health in MCS6 (outcome: having mental health problems, SDQ-TDS ≥ 17; total sample *n* = 10,357 (5298 girls; 5059 boys), weighted *n* = 10,216)

Ethnicity	Model 1: unadjusted		Model 2: basic adjusted for household income		Model 3^a^: overall adjusted model for all social support, participation, adversity factors and household income	
	Girls OR (95% CI)	Boys OR (95% CI)	Girls OR (95% CI)	Boys OR (95% CI)	Girls OR (95% CI)	Boys OR (95% CI)
White						
*n* = 8315 (4257 girls; 4058 boys)	1.00 (ref)	1.00 (ref)	1.00 (ref)	1.00 (ref)	1.00 (ref)	1.00 (ref)
Mixed						
*n* = 492 (246 girls; 246 boys)	1.57 (0.89–2.75)	0.81 (0.48–1.35)	1.35 (0.74–2.47)	0.72 (0.43–1.20)	1.53 (0.85–2.76)	0.77 (0.45, 1.31)
Indian						
*n* = 275 (130 girls; 145 boys)	0.41 (0.13–1.27)	0.42 (0.21–0.86)*	0.38 (0.12–1.22)	0.40 (0.21–0.77)**	0.61 (0.19–2.01)	0.50 (0.25, 1.03)
Pakistani						
*n* = 496 (265 girls; 231 boys)	1.18 (0.77–1.83)	0.91 (0.51–1.64)	0.63 (0.41–0.99)*	0.49 (0.27–0.89)*	0.92 (0.55–1.53)	0.90 (0.46, 1.78)
Bangladeshi						
*n* = 221 (120 girls; 101 boys)	0.34 (0.10–1.23)	1.21 (0.63–2.34)	0.18 (0.05–0.65)**	0.64 (0.34–1.21)	0.31 (0.09–1.11)	1.24 (0.59, 2.59)
Black Caribbean						
*n* = 102 (47 girls; 55 boys)	1.36 (0.43–4.34)	1.86 (0.75–4.64)	0.92 (0.28–3.08)	1.22 (0.45–3.28)	1.30 (0.42–4.09)	1.35 (0.57, 3.21)
Black African						
*n* = 187 (93 girls; 94 boys)	0.43 (0.12–1.57)	0.15 (0.04–0.61)**	0.27 (0.07–1.02)	0.10 (0.02–0.38)**	0.42 (0.09–1.90)	0.16 (0.03, 0.71)*
Other Ethnic Group						
*n* = 269 (140 girls; 129 boys)	1.46 (0.73–2.93)	1.47 (0.75–2.90)	1.03 (0.50–2.10)	1.18 (0.60–2.33)	1.35 (0.66–2.75)	1.51 (0.74, 3.06)
Adjusted Wald test results (association of ethnicity/outcome)						
	F(7, 383) = 1.65, *P* = 0.12	F(7,383) = 2.97, *P* = 0.0048	Ethnicity: F(7, 383) = 2.45, *P* = 0.018 Ethnicity*sex interaction: F(15, 375) = 5.34, *P* < 0.00001		Ethnicity: F(7, 383) = 1.23, *P* = 0.2839 Ethnicity*sex interaction: F(15, 375) = 2.97, *P* = 0.0002	

For individual parameter Wald test results: *P < 0.05, **P < 0.01, ***P < 0.001

^a^Results for all other variables in Model 3 are displayed in Table 3

Across unadjusted logistic regression models of outcome by each social factor, young people in the lowest income quintile had higher odds of having mental health problems compared with those in the highest (OR 4.98, 95% CI 3.55–6.99). Higher odds of mental health problems were also seen in those reporting weaker parental relationships (OR 3.13, 95% CI 1.91–5.13) and spending less time with friends outside school (OR 2.25, 95% CI 1.56–3.25), than those with strong relationships or more frequent social activity, as did those who reported never participating in organised activities (OR 1.37, 95% CI 1.11–1.69) or attending religious services (OR 1.93, 95% CI 1.46–2.54) compared to those who participated in these activities at least weekly, and for those reporting lower levels of social support compared to higher support (OR 4.36, 95% CI 2.42–7.86), frequent bullying (OR 7.46, 95% CI 5.62–9.89) or being a bully (OR 7.11, 95% CI 4.18–12.08) compared to never being a victim or a perpetrator of bullying, and three or more experiences of victimisation (OR 4.06, 95% CI 3.11–5.30) and substance use (OR 6.05, 95% CI 3.58–10.23) compared to no experiences of these, respectively (see Table 3 for full details).

**Table 3:** Association of social support, participation, and adversity with mental health problems in MCS6 (total sample *n* = 10,357, weighted *n* = 10,216)

Social factors	Univariable logistic regression results		Multivariable logistic regression results (model 3, fully adjusted for all other factors, including ethnicity)	
	OR	Adjusted Wald test results, association of factor with outcome	OR	Adjusted Wald test results, association of factor with outcome
Household income				
Highest quintile	1.00 (ref)		1.00 (ref)	
Fourth quintile	1.30 (0.88–1.92)		1.21 (0.81–1.81)	
Third quintile	3.17 (2.23–4.50)		2.71 (1.90–3.86)	
Second quintile	4.56 (3.20–6.50)		3.59 (2.52–5.13)	
Lowest quintile	4.98 (3.55–6.99)	F(4, 386) = 34.76, *P* < 0.0001	4.46 (3.12–6.38)	F(4, 386) = 25.29, *P* < 0.0001
Parental relationship				
Close, both parents	1.00 (ref)		1.00 (ref)	
Close, one parent	2.32 (1.84–2.94)		1.29 (1.001–1.66)	
Not close to parents	3.13 (1.91–5.13)	F(2, 388) = 29.95, *P* < 0.0001	1.09 (0.59–2.00)	F(2,388) = 2.05, *P* = 0.13
Social support				
Strong support	1.00 (ref)		1.00 (ref)	
Some support	2.46 (1.93–3.13)		1.62 (1.24–2.10)	
Little to no support	4.36 (2.42–7.86)	F(2, 388) = 35.44, *P* < 0.0001	2.26 (1.16–4.37)	F(2,388) = 7.79, *P* = 0.0005
Time spent with friends outside school				
Weekly or more	1.00 (ref)		1.00 (ref)	
Monthly or less often	0.98 (0.81–1.19)		1.16 (0.93–1.43)	
Never	2.25 (1.56–3.25)	F(2, 388) = 10.10, *P* = 0.0001	1.80 (1.17–2.76)	F(2, 388) = 3.90, *P* = 0.021
Organised activities				
At least weekly	1.00 (ref)		1.00 (ref)	
Between once a year and once a month	1.25 (0.96–1.64)		1.06 (0.81–1.40)	
Never/almost never	1.37 (1.11–1.69)	F(2, 388) = 4.54, *P* = 0.012	1.06 (0.85–1.31)	F(2,388) = 0.17, *P* = 0.84
Religious attendance				
At least weekly	1.00 (ref)		1.00 (ref)	
Between once a year and once a month	1.12 (0.83–1.51)		1.21 (0.85–1.72)	
Never/almost never	1.93 (1.46–2.54)	F(2, 388) = 17.50, *P* < 0.0001	1.60 (1.14–2.26)	F(2,388) = 4.72, *P* = 0.0095
Experience of bullying				
Never	1.00 (ref)		1.00 (ref)	
Less often	1.28 (1.03–1.59)		1.22 (0.94–1.57)	
Every few months	1.52 (1.06–2.17)		1.28 (0.84–1.96)	
Monthly	2.71 (1.87–3.93)		2.05 (1.37–3.08)	
Weekly	2.74 (2.05–3.66)		2.16 (1.50–3.10)	
Most days	7.46 (5.62–9.89)	F(5, 385) = 45.56, *P* < 0.0001	4.46 (3.02–6.61)	F(5,385) = 13.14, *P* < 0.0001
Perpetration of bullying				
Never	1.00 (ref)		1.00 (ref)	
Less often	1.62 (1.33–1.96)		1.07 (0.85–1.35)	
Every few months	2.21 (1.41–3.48)		1.36 (0.81–2.29)	
Monthly	3.08 (1.78–5.35)		1.28 (0.73–2.27)	
Weekly	3.10 (1.97–4.86)		1.24 (0.76–2.02)	
Most days	7.11 (4.18–12.08)	F(5, 385) = 17.76, *P* < 0.0001	1.59 (0.88–2.87)	F(5,385) = 0.71, *P* = 0.62
Experiences of victimisation				
None	1.00 (ref)		1.00 (ref)	
One	1.45 (1.14–1.83)		1.06 (0.82–1.38)	
Two	2.20 (1.71–2.84)		1.20 (0.91–1.57)	
Three or more	4.06 (3.11–5.30)	F(3, 387) = 40.39, *P* < 0.0001	1.40 (1.00–1.97)	F(3,387) = 1.53, *P* = 0.21
Experiences of substance use				
None	1.00 (ref)		1.00 (ref)	
One	1.57 (1.14–2.17)		1.13 (0.80–1.60)	
Two	3.08 (1.96–4.84)		2.13 (1.36—3.34)	
Three or more	6.05 (3.58–10.23)	F(3, 387) = 27.36, *P* < 0.0001	3.19 (1.84–5.54)	F(3,387) = 9.64, *P* < 0.0001

Estimates displayed in model 3 are adjusted for all displayed variables and an ethnicity*gender interaction (Wald test results for ethnicity*sex interaction: F(15,375) = 2.97, *P* = 0.0002)

On adjustment for household income (model 2, Table 2), odds for having mental health problems were lower in girls from Bangladeshi (OR 0.18, 95% CI 0.05–0.65) and Pakistani (OR 0.63, 95% CI 0.41–0.99) backgrounds, and boys from Black African (OR 0.10, 95% CI 0.02–0.38), Indian (OR 0.40, 95% CI 0.21–0.77), and Pakistani (OR 0.49, 95% CI 0.27–0.89) backgrounds, compared to their White peers. Though confidence intervals for point estimates were wide and overlapping, effect size decreased for those from Other Ethnic Group, boys from Black Caribbean, and girls from Mixed backgrounds; effect size increased for boys from Mixed backgrounds and girls from Black African and Indian backgrounds. Odds were lower for girls from Black Caribbean backgrounds in this model, which was the opposite of the unadjusted findings.

After adjustment for all social support, participation, and adversity factors, together with household income (model 3), only boys from Black African backgrounds had significantly lower odds of having mental health problems compared to White peers (OR 0.16, 95% CI: 0.03–0.71, Table 2). ORs for young people from other backgrounds were largely similar to those obtained in the unadjusted analysis (model 1). There was also a dose–response relationship between greater experiences of bullying (both being victim and perpetrator) and victimisation and poorer mental health (estimates are attenuated on full adjustment, but associations remain).

Logistic regression results are reported here given the rare disease assumption (prevalence below 10%) remains valid for this investigation of mental health outcomes. However, sensitivity analysis repeating the above models using Poisson regression, assessing the SDQ as a linear measure modelling count of symptoms for total score, internalising, and externalising subscales found similar results by ethnicity and sex. Results broadly followed the same direction, however girls from Black African backgrounds in the fully adjusted Poisson regression had a lower total score symptom count, which was not seen from the logistic regression given very wide 95% confidence intervals around the OR. This reflected some improved power in the former approach. In fully adjusted Poisson regression for the internalising subscale, girls from Indian and Black African backgrounds had lower symptom counts, and boys from Pakistani and Other Ethnic Group backgrounds had higher symptom counts compared to White peers, whereas for the externalising subscale, no significant differences were found by ethnicity though the estimates largely followed same patterns at the logistic regression results (see Supplementary Tables 4–6).

## Discussion

Household income was the factor associated with the most substantial changes in effect sizes. The lower odds of having mental health problems in all young people from Pakistani backgrounds and girls from Bangladeshi backgrounds compared to their White peers were seen after adjustment for income (in the unadjusted analysis, Bangladeshi boys and Pakistani girls had higher odds, whereas Bangladeshi girls and Pakistani boys had lower odds, though these estimates had wide confidence intervals). For boys from Indian and Black African backgrounds who had a lower unadjusted odds of having mental health problems, adjusting for income demonstrated a larger effect size; this was also noted in groups for whom lower odds estimates had wide confidence intervals. Similarly, for those who had higher odds estimates with wide confidence intervals, estimates moved closer to the null after adjusting for income. These findings support existing research suggesting ethnic inequalities in mental health are closely related to but not entirely explained by other socioeconomic inequalities [22]. Overall, social support and participation factors were associated with better, and social adversity with worse, mental health outcomes for this nationally representative, ethnically diverse cohort of young people.

There is also very weak evidence of higher prevalence of mental health problems in young people from Black Caribbean and Other Ethnic Group backgrounds, and girls from a Mixed ethnic background. Due to small sample sizes, actual differences remained undetected. These differences are important to investigate further, given that the latest adult data indicate a higher prevalence of common mental disorders in Black and Black British women, and a lower prevalence in White Other women; the prevalence of psychosis is also higher in Black men, compared to White British men [23].

Initial MCS findings at age 14 found that 24% of girls and 9% of boys reported experiencing high levels of depressive symptoms as measured by the Short Moods and Feelings Questionnaire; young people from Asian backgrounds and girls from Black backgrounds were less likely to have high levels of depressive symptoms [24]. Similar results were found by ethnicity in this analysis, however, increased mental health problems were observed in boys here, as indicated by the SDQ, which captures conduct disorders. This difference emphasises the importance of potential mediating mechanisms between gender, social factors, and their impact on mental health outcomes in the transitional period of adolescence, which warrant further investigation (as gender could not be explored beyond the binary sex category available for analysis) [25].

Some of the observed ethnic differences seem to be partly explained by the social factors investigated here, as seen in the estimates in model 3 (adjusted for income and all factors) moving towards the null compared to model 2 (adjusted for income only) for girls from Bangladeshi and all those from Black African, Indian, and Pakistani backgrounds, although confidence intervals overlapped. As differences remained partly but not fully explained, further analyses of mediating mechanisms should be taken to improve understanding. Similar observations have been made in east London, where bullying and social support from families was found to be more important for mental health, whatever the participants’ ethnicity or culturally influenced friendship choices [11]. Experience of racism was an adverse influence, whereas family relationships, religious participation, and ethnically diverse friendships were protective influences for young people from all ethnic backgrounds across London [26].

### Strengths and limitations

This study is the first focused analysis of ethnicity and mental health in a nationally representative cohort of young people, indicating that further investigation of the social factors explored here is needed, as these factors could partly but not fully explain mental health differences observed by ethnicity. Further strengths include generalisability of findings regarding experiences of social support, participation, and adversity.

Despite oversampling to boost ethnic minority representation, the very small sample sizes mean this analysis may be underpowered to detect differences. Ethnic diversity in the UK has changed considerably since 2000, when this birth cohort was recruited. Diverse populations have been homogenised into limited categories which have varying levels of relevance for individuals. The Black African, Black Caribbean, Mixed, and Other Ethnic Groups, amongst others, encompass immense diversity. The White group includes young people from Irish, White Other, and Gypsy or Irish Traveller backgrounds, who have been noted to have different mental health and social outcomes to White British adolescents [12]. Recently migrated or undocumented young people were not included. These findings should be critically appraised in recognition of this complex heterogeneity of cultures, class status, and migration histories [27, 28], as essentialising these differences runs the risk of racial determinism [29, 30].

Processes of socialisation vary between and within cultures, however cultural identity and community specific norms that are underlying the social factors explored here could not be assessed in this analysis. The complex relationship between intergenerational dynamics and mental health was not assessed. While ethnic density (the proportion of people from a given ethnic background resident in a given area) and the related social cohesion this may represent has been associated with lower prevalence of common mental disorders in adults, evidence is mixed for adolescents and was not explored here [31, 32]. It is important to note that patterns of lower prevalence of mental health problems are not maintained into adulthood. The cumulative impact of different forms of adversity and discrimination, including racism over the life-course, interacting with persisting barriers to social mobility may be potentially driving such trends [33, 34]. No questions were directly asked about young people’s experiences of racism in this survey wave; maternal and family experiences of racism were found to affect children’s socioemotional development for this cohort [35]. Monitoring changes across the life-course is important, moreover as compared to White British peers, ethnic minority children and young people are more likely to access mental health services through compulsory routes, reflecting inequalities found in adults [36].

All measures were mostly self-reported by young people and parents, so were subject to reporting bias. A key limitation was lack of young people’s self-reported SDQ responses, to compare and corroborate results with parent reports given their respective biases. Despite widespread application across international contexts, evidence remains limited on SDQ construct validity for adolescents across cultures and languages in the UK context (with only one study reviewing it as valid in adolescents from Indian backgrounds) [37] and whether parental willingness to report negative behaviours is associated with ethnicity, given mental health related stigma reported in ethnic minority groups [38]. The SDQ was not systematically translated for those with limited English, and differences in idioms of mental distress across populations may not have been captured [21, 39].

Cross-sectional analysis of a single sweep of a longitudinal study meant that the temporal sequence of risk factors could not be established. Furthermore, in light of the recent unequal impact of the COVID-19 pandemic and response measures taken in the UK, which has resulted in a disproportionately greater loss of income in ethnic minority groups who were already overrepresented in disadvantage, monitoring income inequalities is particularly important [40]. In future, there will be a potential to address these limitations and follow the same participants, in the age 17 and 22 sweeps of the MCS.

## Conclusions

Household income confounded the lower prevalence of mental health problems apparent in some young people from Bangladeshi and Pakistani backgrounds compared to White peers; lower prevalence was also noted in some young people from Black African and Indian backgrounds, for whom effect sizes were stronger after adjusting for household income. There was some evidence of a higher prevalence of mental health problems in young people from Black Caribbean, Mixed, and Other Ethnic Group backgrounds which should be investigated further, given patterns of ethnic inequalities in mental health observed in adulthood.

These findings suggest these ethnic inequalities in mental health outcomes could be partly but not fully explained by social support, participation, and adversity factors. Further analysis of mediating mechanisms and differences by gender should be closely monitored. For example, where young people find social support is often also where they experience social adversity [41]: supportive friendships can turn into sources of risk if peer groups encourage risky behaviours, and the adolescent-parent relationship can be a source of stress. Social participation is increasingly conducted online; while only negative influences of social media in cyberbullying was considered here, the role of social media in facilitating potentially beneficial social interactions for wellbeing could be investigated further.

Overall, interventions that seek to tackle household income inequalities (such as improved housing and financial support for struggling families) and prevent bullying and victimisation in particular (for example across school, community, and social media settings) may support young people against mental health problems, irrespective of ethnicity.

## Supplementary Information

Below is the link to the electronic supplementary material.

Supplementary file1 (DOCX 76 KB)

## Data Availability

MCS6 is deposited with the UK Data Service at the University of Essex.

## Code availability

Analysis code can be made available on request.
