# Supplementary material for: Explaining ethnic variations in adolescent mental health: a secondary analysis of the Millennium Cohort Study
Source: Soc Psychiatry Psychiatr Epidemiol. 2021 Oct 24;57(4):817–28. doi: 10.1007/s00127-021-02167-w (PMC8541880; doi:10.1007/s00127-021-02167-w)
Supplement: Supplementary file 1 — Supplementary file1 (DOCX 76 KB) [file 127_2021_2167_MOESM1_ESM.docx]

**Explaining ethnic variations in adolescent mental health: a secondary analysis of the Millennium Cohort Study**

**Gargie Ahmad^1^, Sally McManus^2^, Laia Bécares^3^, Stephani Hatch^1^, Jayati Das-Munshi^1^**

**Supplementary material for Social Psychiatry and Psychiatric Epidemiology submission**

1. King’s College London
2. City, University of London, and National Centre for Social Research
3. University of Sussex

Corresponding author: Gargie Ahmad, [gargie.ahmad@kcl.ac.uk](mailto:gargie.ahmad@kcl.ac.uk)

List of supplementary tables:

Table S1 Prevalence of mental health problems (SDQ TDS ≥17) by all factors in Millennium Cohort Study Sweep 6 (total complete case sample n=10,357, weighted n=10,216)

Table S2: Distribution of demographic and social factors by ethnicity in MCS6 for girls (n=5298, weighted column proportions, %)

Table S3: Distribution of demographic and social factors by ethnicity in MCS6 for boys (n=5059, weighted column proportions, %)

Table S4: Poisson regression results summary of association between ethnicity and mental health in MCS6

Outcome: SDQ total scores scale

Table S5: Poisson regression results summary of association between ethnicity and mental health in MCS6

Outcome: SDQ internalising subscale

Table S6: Poisson regression results summary of association between ethnicity and mental health in MCS6

Outcome: SDQ externalising subscale

| **Table S1: Prevalence of mental health problems (SDQ TDS ≥17) by all factors in Millennium Cohort Study Sweep 6  (total complete case sample n=10,357, weighted n=10,216)** | | | | | | | |
| --- | --- | --- | --- | --- | --- | --- | --- |
| **Factors** | **Case n** | **Prevalence**  **(weighted %,**  **95% CI)** | **Χ2 adjusted**  **F (P)** | **Factors** | **Case n** | **Prevalence**  **(weighted %, 95% CI)** | **Χ2 adjusted**  **F (P)** |
| **Whole sample** (n=10357) | 849 | **9.9 (9.1-10.6)** |  | ***Time spent in religious attendance*** | |  |  |
| ***Ethnicity*** |  |  |  | **Never/almost never** (n=5557) | 545 | **11.9 (10.6-13.2)** |  |
| **White** (n=8315) | 665 | **10.0 (9.0-11.0)** |  | **Between once a year and once a month** (n=3163) | 197 | **7.2 (6.0-8.6)** |  |
| **Mixed** (n=492) | 49 | **11.0 (8.0-14.6)** |  | **At least weekly** (n=1637) | 107 | **6.5 (5.2-8.2)** | 20.6 (P<0.0001) |
| **Indian** (n=275) | 16 | **4.5 (2.5-7.8)** |  | ***Social support*** |  |  |  |
| **Pakistani** (n=496) | 55 | **10.2 (8.1-12.9)** |  | **Little/no social support** (n=117) | 25 | **28.7 (18.4-41.8)** |  |
| **Bangladeshi** (n=221) | 15 | **8.0 (5.9-10.7)** |  | **Some social support** (n=1147) | 174 | **18.5 (15.7-21.7)** |  |
| **Black Caribbean** (n=102) | 13 | **15.8 (8.8-26.9)** |  | **Strong social support** (n=9093) | 650 | **8.5 (7.6-9.4)** | 39.2 (P<0.0001) |
| **Black African** (n=187) | 5 | **2.9 (1.1-7.3)** |  | ***Being a victim of bullying*** |  |  |  |
| **Other Ethnic Group** (n=269) | 31 | **14.0 (9.0-21.3)** | 3.4 (P=0.0040) | **Never** (n=4549) | 251 | **6.7 (5.7-7.7)** |  |
| ***Sex*** |  |  |  | **Less often** (n=3413) | 244 | **8.4 (7.2-9.7)** |  |
| **Female** (n=5298) | 404 | **9.0 (8.0-10.0)** |  | **Every few months** (n=721) | 62 | **9.8 (7.3-12.9)** |  |
| **Male** (n=5059) | 445 | **10.9 (9.6-12.2)** | 6.4 (P=0.012) | **About once a month** (n=602) | 74 | **16.2 (12.2-21.2)** |  |
| ***Household income*** |  |  |  | **About once a week** (n=642) | 99 | **16.3 (13.2-20.1)** |  |
| **Lowest quintile** (n=1583) | 236 | **16.4 (14.1-18.9)** |  | **Most days** (n=430) | 119 | **34.7 (29.7-40.0)** | 55.6 (P<0.0001) |
| **Second quintile** (n=1703) | 219 | **15.2 (12.7-18.0)** |  | ***Being a perpetrator of bullying*** | |  |  |
| **Third quintile** (n=2127) | 195 | **11.1 (9.4-13.0)** |  | **Never** (n=6868) | 460 | **7.7 (6.9-8.6)** |  |
| **Fourth quintile** (n=2489) | 112 | **4.9 (3.7-6.3)** |  | **Less often** (n=2595) | 257 | **11.8 (10.3-13.5)** |  |
| **Highest quintile** (n=2455) | 87 | **3.8 (2.8-5.0)** | 36.7 (P<0.0001) | **Every few months** (n=337) | 38 | **15.5 (10.5-22.3)** |  |
| ***Parental relationship*** |  |  |  | **About once a month** (n=240) | 29 | **20.4 (12.8-30.8)** |  |
| **Not very close to either/no parents** (n=162) | 33 | **21.3 (14.5-30.2)** |  | **About once a week** (n=219) | 33 | **20.4 (14.1-28.6)** |  |
| **Close to one parent** (n=1662) | 238 | **16.7 (14.1-19.7)** |  | **Most days** (n=98) | 32 | **37.1 (25.9-49.8)** | 20.2 (P<0.0001) |
| **Close to both parents** (n=8533) | 578 | **8.0 (7.1-8.7)** | 35.8, (P<0.0001) | ***Experiences of victimisation*** |  |  |  |
| ***Time spent with friends outside school*** | |  |  | **None** (n=5332) | 320 | **6.9 (6.0-8.0)** |  |
| **Never** (n=341) | 55 | **19.4 (14.5-25.4)** |  | **One** (n=2850) | 220 | **9.7 (8.1-11.7)** |  |
| **Monthly** (n=2527) | 200 | **9.5 (8.1-11.1)** |  | **Two** (n=1556) | 188 | **14.1 (11.8-16.8)** |  |
| **Weekly or more** (n=7489) | 594 | **9.6 (8.7-10.7)** | 10.8 (P<0.0001) | **Three or more** (n=619) | 121 | **23.2 (19.3-27.7)** | 37.5 (P<0.0001) |
| ***Time spent in organised activities*** | |  |  | ***Experiences of substance abuse*** | |  |  |
| **Never/almost never** (n=4370) | 412 | **11.1 (9.7-12.6)** |  | **None** (n=9169) | 675 | **8.7 (7.9-9.6)** |  |
| **Between once a year and once a month** (n=2027) | 163 | **10.2 (8.4-12.4)** |  | **One** (n=824) | 91 | **13.0 (10.0-16.9)** |  |
| **At least weekly** (n=3960) | 274 | **8.3 (7.2-9.5)** | 4.3 (P=0.014) | **Two** (n=253) | 46 | **22.7 (16.0-31.2)** |  |
|  |  |  |  | **Three or more** (n=111) | 37 | **36.6 (25.2-49.8)** | 26.7 (P<0.0001) |
|  |  |  |  |  |  |  |  |

| **Table S2: Distribution of demographic and social factors by ethnicity in MCS6 for girls (n=5298, weighted column proportions, %)** | | | | | | | | | | | | |
| --- | --- | --- | --- | --- | --- | --- | --- | --- | --- | --- | --- | --- |
| **Ethnicity** | | **White (n=4257** | | **Mixed**  **(n=246)** | **Indian**  **(=130)** | **Pakistani (n=265)** | **Bangladeshi (n=120)** | **Black Caribbean (n=47)** | **Black African (n=93)** | **Other Ethnic Group (n=140)** | **Total, girls (n=5298)** | **Chi-squared adjusted design-based F** |
| **Prevalence of mental health problems (SDQ TDS ≥17)** | | | | | | | | | | | | |
| n  %  (95% CI) | | 314  8.8  (7.8-10.1) | | 27  13.2  (8.2-20.7) | 8  3.8  (1.3-10.8) | 30  10.3  (7.1-14.8) | 3  3.2  (0.9-10.6) | 5  11.7  (4.1-29.1) | 3  4.0  (1.1-13.1) | 14  12.4  (6.7-21.9) | 404  9.0  (8.0-10.0) | 1.7  P=0.1223 |
| **Distribution of social factors by ethnicity, n (%)** | | | | | | | | | | | | |
| **Household income** | | | | | | | | | | | | |
| **Lowest quintile** | | 430 (13.3) | | 51 (22.8) | 10 (7.21) | 169 (64.9) | 80 (70.7) | 10 (31.1) | 38 (47.5) | 49 (40.1) | 837 (17.9) |  |
| **Second quintile** | | 669 (19.1) | | 48 (21.7) | 32 (30.6) | 74 (26.8) | 29 (22.1) | 16 (34.8) | 19 (17.2) | 25 (19.4) | 912 (19.9) |  |
| **Third quintile** | | 913 (21.4) | | 51 (20.2) | 32 (23.7) | 15 (5.49) | 8 (5.76) | 10 (17.2) | 19 (20.5) | 27 (17.5) | 1075 (20.5) |  |
| **Fourth quintile** | | 1112 (22.6) | | 45 (15.6) | 28 (16.6) | 6 (2.6) | 3 (1.4) | 8 (12.6) | 9 (7.3) | 28 (16.1) | 1239 (20.5) |  |
| **Highest quintile** | | 1133 (23.5) | | 51 (19.9) | 28 (21.8) | 1 (0.1) | 0 (0) | 3 (4.3) | 8 (7.6) | 11 (6.9) | 1235 (21.1) | 15.5  P<0.0001 |
| **Parental relationship** | | | | | | | | | | | | |
| **Not very close to either/no parents** | | 79 (2.4) | | 9 (4.6) | 4 (2.8) | 8 (2.8) | 4 (3.4) | 4 (13.8) | 3 (3.1) | 3 (2.6) | 114 (2.7) |  |
| **Close to one parent** | | 769 (22) | | 66 (29.6) | 15 (13) | 49 (17.1) | 16 (12.7) | 16 (30.3) | 15 (17.6) | 22 (23) | 968 (22) |  |
| **Close to both parents** | | 3409 (75.5) | | 171 (65.8) | 111 (84.2) | 208 (80.1) | 100 (84) | 27 (55.8) | 75 (79.4) | 115 (74.4) | 4216 (75.3) | 2.8  P=0.0017 |
| **Social support** | | | | | | | | | | | | |
| **Little/no social support** | | 52 (1.5) | | 6 (1.9) | 2 (0.7) | 8 (2.8) | 2 (1.6) | 2 (8.8) | 1 (1.8) | 4 (4.6) | 77 (1.7) |  |
| **Some social support** | | 439 (11.3) | | 40 (17) | 14 (12) | 30 (11) | 10 (7.6) | 8 (14.3) | 9 (13.2) | 20 (11.6) | 570 (11.6) |  |
| **Strong social support** | | 3766 (87.2) | | 200 (81.1) | 114 (87.3) | 227 (86.2) | 108 (90.8) | 37 (76.9) | 83 (85.1) | 116 (83.8) | 4651 (86.7) | 1.9  P=0.505 |
| **Time spent with friends outside school** | | | | | | | | | | | | |
| **Never** | | 55 (1.5) | | 16 (6.2) | 10 (5.8) | 49 (19.7) | 18 (15) | 2 (6) | 3 (4) | 13 (8.7) | 166 (2.9) |  |
| **Monthly** | | 966 (21.6) | | 68 (25.8) | 71 (51.6) | 97 (33.3) | 61 (44.8) | 16 (31.7) | 40 (36.7) | 54 (41.5) | 1373 (24.1) |  |
| **Weekly or more** | | 3236 (76.9) | | 162 (68) | 49 (42.6) | 119 (47) | 41 (40.1) | 29 (62.3) | 50 (59.3) | 73 (49.7) | 3759 (73) | 20.1  P<0.0001 |
| **Time spent in organised activities** | | | | | | | | | | | | |
| **Never/almost never** | | 1799 (46.3) | | 111 (47.1) | 51 (36.1) | 155 (61.9) | 60 (48.2) | 13 (27) | 40 (45.9) | 56 (48.3) | 2285 (46.5) |  |
| **Between once a year-once a month** | | 773 (18.7) | | 48 (19.2) | 43 (33.9) | 71 (22.7) | 39 (30.7) | 13 (31) | 30 (28.9) | 39 (28.3) | 1056 (19.9) |  |
| **At least weekly** | | 1685 (35) | | 87 (33.6) | 36 (30) | 39 (15.4) | 21 (21.1) | 21 (41.9) | 23 (25.2) | 45 (23.4) | 1957 (33.5) | 4.0  P<0.0001 |
|  | |  | |  |  |  |  |  |  |  | Table S2 continued overleaf | |
| **Table S2 continued: Distribution of demographic and social factors by ethnicity in MCS6 for girls (n=5298, weighted column proportions, %)** | | | | | | | | | | | | |
| **Ethnicity**  **Factor** | **White (n=4257)** | | | **Mixed**  **(n=246)** | **Indian**  **(=130)** | **Pakistani (n=265)** | **Bangladeshi (n=120)** | **Black Caribbean (n=47)** | **Black African (n=93)** | **Other Ethnic Group (n=140)** | **Total, girls (n=5298)** | **Chi-squared adjusted design-based F** |
| **Time spent in religious attendance** | | |  |  |  |  |  |  |  |  |  |  |
| Never/almost never | 2433 (62.8) | | | 108 (50.9) | 18 (16.7) | 35 (14.7) | 27 (21.9) | 13 (24.6) | 7 (11.1) | 45 (36.2) | 2686 (56.8) |  |
| Between once a year-once a month | 1346 (29.6) | | | 87 (32.6) | 79 (57.5) | 115 (38.7) | 54 (43.9) | 20 (43.6) | 19 (16) | 55 (36.7) | 1775 (30.9) |  |
| At least weekly | 478 (7.6) | | | 51 (16.5) | 33 (25.7) | 115 (46.6) | 39 (34.2) | 14 (31.8) | 67 (72.9) | 40 (27.2) | 837 (12.3) | 39.7  P<0.0001 |
| **Being a victim of bullying** | | | | | | | | | | | | |
| **Never** | 1665 (38.5) | | | 110 (44.7) | 63 (53.1) | 148 (56.8) | 66 (54.9) | 24 (57.8) | 56 (51.7) | 74 (47.6) | 2206 (40.7) |  |
| **Less often** | 1504 (35.1) | | | 85 (35.9) | 48 (34.7) | 70 (25.9) | 41 (36.2) | 15 (29.4) | 27 (34.7) | 46 (36.7) | 1836 (34.8) |  |
| **Every few months** | 332 (7.7) | | | 19 (7) | 7 (5.4) | 10 (2.7) | 4 (3.5) | 4 (6.1) | 4 (5.6) | 5 (3.3) | 385 (7.2) |  |
| **About once a month** | 293 (6.8) | | | 14 (5.1) | 5 (2.9) | 9 (4.3) | 4 (2.4) | 1 (3.4) | 3 (1.7) | 4 (3.8) | 333 (6.2) |  |
| **About once a week** | 277 (6.7) | | | 6 (1.8) | 5 (2.8) | 11 (4.4) | 2 (1.5) | 2 (2.1) | 2 (1.5) | 9 (7.8) | 314 (6.1) |  |
| **Most days** | 186 (5.3) | | | 12 (5.5) | 2 (1.1) | 17 (5.9) | 3 (1.6) | 1 (1.2) | 1 (4.8) | 2 (0.8) | 224 (5) | 2.2  P=0.0013 |
| **Being a perpetrator of bullying** | | | | | | | | | | | | |
| **Never** | 2966 (68) | | | 167 (68.3) | 95 (74.6) | 192 (76.3) | 82 (69.5) | 32 (65.2) | 64 (62.8) | 81 (51.6) | 3679 (67.8) |  |
| **Less often** | 1012 (25.1) | | | 54 (20.8) | 25 (15.4) | 52 (16.3) | 31 (25.8) | 8 (15.9) | 20 (22.6) | 48 (37.9) | 1250 (24.6) |  |
| **Every few months** | 117 (2.8) | | | 8 (4.4) | 4 (5.7) | 4 (1.3) | 1 (0.6) | 3 (8.7) | 2 (2.6) | 4 (4.1) | 143 (2.9) |  |
| **About once a month** | 85 (2) | | | 6 (1.9) | 2 (0.7) | 4 (2.2) | 1 (0.4) | 2 (8.1) | 3 (2.1) | 0 (0) | 103 (2) |  |
| **About once a week** | 58 (1.4) | | | 5 (1.9) | 4 (3.6) | 8 (2.4) | 4 (3) | 1 (0.9) | 3 (8.3) | 4 (2.4) | 87 (1.7) |  |
| **Most days** | 19 (0.6) | | | 6 (2.7) | 0 (0) | 5 (1.4) | 1 (0.7) | 1 (1.2) | 1 (1.7) | 3 (4) | 36 (0.9) | 2.6  P=0.0001 |
| **Experiences of victimisation** | | | | | | | | | | | | |
| **None** | 2146 (48.8) | | | 133 (47.9) | 83 (69.5) | 175 (69.1) | 79 (70.3) | 24 (60.8) | 56 (55.6) | 91 (62.7) | 2787 (50.9) |  |
| **One** | 1344 (31.9) | | | 74 (37) | 35 (25) | 56 (20.5) | 32 (22.6) | 14 (24.2) | 26 (30.5) | 35 (26.3) | 1616 (31.2) |  |
| **Two** | 538 (12.8) | | | 26 (11.4) | 10 (4.5) | 24 (7.7) | 9 (7.2) | 6 (9.8) | 5 (4.6) | 8 (6.7) | 626 (12) |  |
| **Three or more** | 229 (6.4) | | | 13 (3.7) | 2 (1) | 10 (2.7) | 0 (0) | 3 (5.2) | 6 (9.4) | 6 (4.2) | 269 (5.9) | 3.0  P=0.0006 |
| **Experiences of substance use** | | | | | | | | | | | | |
| **None** | 3714 (85) | | | 217 (85.5) | 128 (98.3) | 260 (98.1) | 120 (100) | 42 (84.7) | 91 (97.8) | 130 (94.3) | 4702 (86.4) |  |
| **One** | 370 (9.9) | | | 18 (9.2) | 1 (0.3) | 4 (1.6) | 0 (0) | 3 (10.4) | 1 (0.5) | 5 (2) | 402 (8.8) |  |
| **Two** | 116 (3.5) | | | 8 (4.7) | 1 (1.4) | 0 (0) | 0 (0) | 2 (4.8) | 1 (1.7) | 3 (2.7) | 131 (3.3) | 1.7  P=0.0896 |
| **Three or more** | 57 (1.7) | | | 3 (0.6) | 0 (0) | 1 (0.3) | 0 (0) | 0 (0) | 0 (0) | 2 (1.1) | 63 (1.4) |  |

| **Table S3: Distribution of demographic and social factors by ethnicity in MCS6 for boys (n=5059, weighted column proportions, %)** | | | | | | | | | | | | |
| --- | --- | --- | --- | --- | --- | --- | --- | --- | --- | --- | --- | --- |
| **Ethnicity** | | **White (n=4058)** | | **Mixed**  **(n=246)** | **Indian**  **(=145)** | **Pakistani (n=231)** | **Bangladeshi (n=101)** | **Black Caribbean (n=55)** | **Black African (n=94)** | **Other Ethnic Group (n=129)** | **Total, boys (n=5059)** | **Chi-squared adjusted design-based F** |
| **Prevalence of mental health problems (SDQ TDS ≥17)** | | | | | | | | | | | | |
| n  %  (95% CI) | | 351  11.1  (9.7-12.7) | | 22  9.1  (5.8-14.2) | 8  5.0  (2.5-9.6) | 25  10.2  (6.1-16.5) | 12  13.1  (7.3-22.4) | 8  18.9  (8.7-36.1) | 2  1.9  (0.5-7.1) | 17  15.5  (8.7-26.2) | 445  10.9  (9.6-12.2) | 2.4  P=0.0284 |
| **Distribution of social factors by ethnicity** | | | | | | | | | | | | |
| **Household income** | | | | | | | | | | | | |
| **Lowest quintile** | | 391 (12.9) | | 43 (18.4) | 10 (10.6) | 155 (63.3) | 65 (69.6) | 21 (46.8) | 35 (47.4) | 26 (23.9) | 746 (17.1) |  |
| **Second quintile** | | 593 (18.5) | | 47 (20.1) | 28 (23.7) | 48 (20.5) | 23 (19.3) | 7 (14) | 18 (24.1) | 27 (22.7) | 791 (18.9) |  |
| **Third quintile** | | 879 (21.2) | | 48 (21.4) | 32 (18.5) | 22 (14.8) | 9 (4.7) | 13 (22.7) | 16 (8.6) | 33 (22.5) | 1052 (20.5) |  |
| **Fourth quintile** | | 1107 (23.7) | | 49 (19.2) | 39 (29.2) | 5 (1.4) | 2 (3.7) | 10 (13.4) | 16 (11.2) | 22 (19) | 1250 (22.1) |  |
| **Highest quintile** | | 1088 (23.7) | | 59 (20.8) | 36 (18.1) | 1 (0.2) | 2 (2.8) | 4 (3.2) | 9 (8.7) | 21 (12) | 1220 (21.5) | 11.5  P<0.0001 |
| **Parental relationship** | | | | | | | | | | | | |
| **Not very close to either/no parents** | | 36 (1.1) | | 5 (2.5) | 1 (1.3) | 3 (1.1) | 0 (0) | 0 (0) | 2 (3.3) | 1 (0.3) | 48 (1.2) |  |
| **Close to one parent** | | 532 (16.2) | | 45 (21.6) | 17 (13) | 32 (14.2) | 9 (8.4) | 16 (46) | 21 (27.2) | 22 (21.4) | 694 (17.1) |  |
| **Close to both parents** | | 3490 (82.7) | | 196 (75.9) | 127 (85.7) | 196 (84.7) | 92 (91.6) | 39 (54) | 71 (69.6) | 106 (78.3) | 4317 (81.7) | 2.5  P=0.0091 |
| **Social support** | | | | | | | | | | | | |
| **Little/no social support** | | 31 (0.7) | | 5 (1) | 2 (1.2) | 1 (0.2) | 0 (0) | 0 (0) | 0 (0) | 1 (0.3) | 40 (0.7) |  |
| **Some social support** | | 443 (12.4) | | 29 (10.6) | 15 (12.8) | 28 (12.3) | 14 (12.1) | 10 (23.3) | 12 (15.9) | 26 (19.6) | 577 (12.8) |  |
| **Strong social support** | | 3584 (86.8) | | 212 (88.4) | 128 (86) | 202 (87.6) | 87 (87.9) | 45 (76.7) | 82 (84.1) | 102 (80.1) | 4442 (86.5) | 0.7  P=0.6571 |
| **Time spent with friends outside school** | | | | | | | | | | | | |
| **Never** | | 116 (3.1) | | 6 (1.4) | 6 (4) | 23 (8.1) | 10 (11.4) | 1 (0.5) | 3 (3.5) | 10 (6.4) | 175 (3.3) |  |
| **Monthly** | | 896 (21) | | 46 (17.3) | 47 (28.4) | 66 (26.5) | 26 (21.8) | 12 (28.6) | 26 (27) | 35 (21.7) | 1154 (21.4) |  |
| **Weekly or more** | | 3046 (75.9) | | 194 (81.4) | 92 (67.6) | 142 (65.4) | 65 (66.8) | 42 (70.9) | 65 (69.5) | 84 (72) | 3730 (75.3) | 2.2  P=0.0234 |
| **Time spent in organised activities** | | | | | | | | | | | | |
| **Never/almost never** | | 1699 (45.7) | | 96 (42.1) | 66 (46.7) | 99 (44.6) | 38 (32.5) | 16 (23.2) | 26 (37.5) | 45 (26.8) | 2085 (44.3) |  |
| **Between once a year-once a month** | | 712 (17.2) | | 51 (18.4) | 33 (19.7) | 68 (28.3) | 34 (29.5) | 16 (43) | 27 (24.7) | 30 (24.5) | 971 (18.6) |  |
| **At least weekly** | | 1647 (37.1) | | 99 (39.5) | 46 (33.6) | 64 (27.1) | 29 (38) | 23 (33.9) | 41 (37.8) | 54 (48.7) | 2003 (37.2) | 3.1  P=0.0005 |
|  | |  | |  |  |  |  |  |  |  | Table S3 continued overleaf | |
| **Table S3 continued: Distribution of demographic and social factors by ethnicity in MCS6 for boys (n=5059, weighted column proportions, %)** | | | | | | | | | | | | |
| **Ethnicity**  **Factor** | **White (n=4058)** | | | **Mixed**  **(n=246)** | **Indian**  **(=145)** | **Pakistani (n=231)** | **Bangladeshi (n=101)** | **Black Caribbean (n=55)** | **Black African (n=94)** | **Other Ethnic Group (n=129)** | **Total, boys (n=5059)** | **Chi-squared adjusted design-based F** |
| **Time spent in religious attendance** | | |  |  |  |  |  |  |  |  |  |  |
| Never/almost never | 2630 (71.2) | | | 132 (57.4) | 23 (17.8) | 10 (4.6) | 7 (6.5) | 21 (53.1) | 7 (7) | 41 (37.6) | 2871 (63.6) |  |
| Between once a year-once a month | 1051 (22.6) | | | 77 (29.3) | 73 (48.9) | 68 (29.3) | 28 (24.4) | 24 (30) | 22 (30.9) | 45 (22.1) | 1388 (24.1) |  |
| At least weekly | 377 (6.2) | | | 37 (13.4) | 49 (33.3) | 153 (66) | 66 (69.2) | 10 (17) | 65 (62.1) | 43 (40.3) | 800 (12.4) | 52.5  P<0.0001 |
| **Being a victim of bullying** | | | | | | | | | | | | |
| **Never** | 1774 (44.3) | | | 113 (48.7) | 89 (65.8) | 150 (67) | 63 (63.4) | 33 (62.8) | 56 (67.5) | 65 (58.3) | 2343 (47.2) |  |
| **Less often** | 1316 (32) | | | 67 (27.8) | 40 (20) | 56 (21.3) | 29 (26.8) | 12 (13) | 21 (15.7) | 36 (26.8) | 1577 (30.2) |  |
| **Every few months** | 285 (6.5) | | | 15 (5.5) | 5 (4.4) | 8 (3.3) | 4 (2.6) | 4 (9.1) | 4 (5.6) | 11 (5.7) | 336 (6.2) |  |
| **About once a month** | 233 (6) | | | 15 (5.8) | 3 (2.2) | 4 (1.6) | 1 (0.8) | 4 (10.5) | 4 (2.7) | 5 (2.5) | 269 (5.6) |  |
| **About once a week** | 273 (6.5) | | | 25 (8.1) | 5 (3.4) | 9 (4.7) | 2 (3.9) | 2 (4.8) | 6 (6.7) | 6 (3.4) | 328 (6.3) |  |
| **Most days** | 177 (4.8) | | | 11 (4.2) | 3 (4.2) | 4 (2.2) | 2 (2.5) | 0 (0) | 3 (1.9) | 6 (3.3) | 206 (4.5) | 2.3  P=0.0010 |
| **Being a perpetrator of bullying** | | | | | | | | | | | | |
| **Never** | 2562 (62.7) | | | 135 (53.3) | 93 (60.2) | 146 (63.7) | 69 (72.3) | 32 (61) | 65 (64.5) | 87 (66.9) | 3189 (62.4) |  |
| **Less often** | 1080 (26.8) | | | 66 (29.8) | 42 (32) | 67 (28.6) | 25 (19) | 17 (25.7) | 20 (26.3) | 28 (22.6) | 1345 (26.9) |  |
| **Every few months** | 156 (3.6) | | | 14 (5.7) | 3 (1.4) | 7 (2.5) | 3 (1.8) | 3 (2) | 4 (7.1) | 4 (1.6) | 194 (3.6) |  |
| **About once a month** | 100 (2.8) | | | 15 (4.8) | 3 (1.8) | 5 (2.1) | 2 (2.5) | 2 (9.6) | 3 (1.1) | 7 (6.8) | 137 (3.1) |  |
| **About once a week** | 108 (2.7) | | | 12 (4.6) | 2 (1.6) | 4 (1.5) | 2 (4.4) | 1 (1.7) | 2 (0.9) | 1 (1.3) | 132 (2.7) |  |
| **Most days** | 52 (1.4) | | | 4 (1.8) | 2 (3) | 2 (1.5) | 0 (0) | 0 (0) | 0 (0) | 2 (0.9) | 62 (1.4) | 1.0  P=0.4183 |
| **Experiences of victimisation** | | | | | | | | | | | | |
| **None** | 1956 (48.4) | | | 105 (41.1) | 94 (64.2) | 162 (71) | 76 (78.5) | 28 (52.3) | 45 (59.6) | 79 (55.9) | 2545 (49.9) |  |
| **One** | 1047 (25) | | | 59 (25.3) | 25 (17) | 34 (15.1) | 12 (10.5) | 12 (14.7) | 25 (19.1) | 20 (17.1) | 1234 (23.9) |  |
| **Two** | 767 (18.6) | | | 54 (23.6) | 19 (13.6) | 30 (12.2) | 10 (6.7) | 13 (30.9) | 19 (18.4) | 18 (18.1) | 930 (18.6) |  |
| **Three or more** | 288 (8) | | | 28 (10) | 7 (5.1) | 5 (1.7) | 3 (4.3) | 2 (2.2) | 5 (2.9) | 12 (9) | 350 (7.6) | 2.9  P=0.0005 |
| **Experiences of substance use** | | | | | | | | | | | | |
| **None** | 3547 (86.5) | | | 206 (85.8) | 142 (95.7) | 225 (97.2) | 97 (97.1) | 47 (85) | 85 (92.4) | 118 (88.7) | 4467 (87.3) |  |
| **One** | 372 (9.2) | | | 23 (8.5) | 0 (0) | 4 (1.3) | 4 (3) | 5 (4.8) | 9 (7.6) | 5 (5.8) | 422 (8.5) |  |
| **Two** | 100 (2.7) | | | 12 (4) | 1 (1.3) | 2 (1.5) | 0 (0) | 3 (10.2) | 0 (0) | 4 (3.4) | 122 (2.7) | 1.4  P=0.1770 |
| **Three or more** | 39 (1.6) | | | 5 (1.7) | 2 (3) | 0 (0) | 0 (0) | 0 (0) | 0 (0) | 2 (2) | 48 (1.5) |  |

| **Table S4: Poisson regression results summary of association between ethnicity and mental health in MCS6**  **Outcome: SDQ total scores scale; total sample n=10,357 (5298 girls; 5059 boys), weighted n=10,216)** | | | | | | |
| --- | --- | --- | --- | --- | --- | --- |
| **Ethnicity** | **Model 1: Unadjusted** | | **Model 2: Basic adjusted for household income** | | **Model 3: Overall adjusted model for all social support, participation, adversity factors and household income** | |
|  | **Girls**  **IRR (95% CI)** | **Boys IRR (95% CI)** | **Girls**  **IRR (95% CI)** | **Boys IRR (95% CI)** | **Girls**  **IRR (95% CI)** | **Boys IRR (95% CI)** |
| **White** n=8315 (4257 girls; 4058 boys) | 1.00 (ref) | 1.00 (ref) | 1.00 (ref) | 1.00 (ref) | 1.00 (ref) | 1.00 (ref) |
| **Mixed**  n=492 (246 girls; 246 boys) | 1.07 (0.95-1.21) | 0.94 (0.84-1.05) | 1.01 (0.90-1.14) | 0.90 (0.81-1.01) | 1.03 (0.92-1.15) | 0.93 (0.83-1.03) |
| **Indian**  n=275 (130 girls; 145 boys) | 0.74 (0.61-0.91)* | 0.96 (0.85-1.08) | 0.73 (0.58-0.90)** | 0.95 (0.86-1.05) | 0.83 (0.67-1.01) | 1.04 (0.94-1.16) |
| **Pakistani**  n=496 (265 girls; 231 boys) | 1.17 (1.07-1.27)** | 1.14 (1.02-1.27)* | 0.91 (0.83-0.99) | 0.89 (0.80-1.00) | 1.02 (0.93-1.11) | 1.07 (0.95-1.21) |
| **Bangladeshi**  n=221 (120 girls; 101 boys) | 1.02 (0.91-1.15) | 1.05 (0.86-1.28) | 0.79 (0.70-0.89)*** | 0.82 (0.68-0.99)* | 0.92 (0.81-1.05) | 0.99 (0.82-1.21) |
| **Black Caribbean**  n=102 (47 girls; 55 boys) | 1.12 (0.90-1.38) | 1.27 (1.09-1.49)** | 0.96 (0.77-1.19) | 1.08 (0.92-1.27) | 1.04 (0.85-1.27) | 1.12 (0.98-1.28) |
| **Black African**  n=187 (93 girls; 94 boys) | 0.84 (0.74-0.95)* | 0.95 (0.80-1.13) | 0.71 (0.64-0.79)*** | 0.80 (0.69-0.93)** | 0.82 (0.72-0.93)** | 0.94 (0.81-1.08) |
| **Other Ethnic Group**  n=269 (140 girls; 129 boys) | 1.13 (0.97-1.31) | 1.20 (1.01-1.42)* | 0.99 (0.86-1.13) | 1.10 (0.93-1.30) | 1.06 (0.92-1.21) | 1.17 (1.00-1.38) |
| Adjusted Wald test results (association of ethnicity/outcome): | | | | | | |
|  | F(7, 383)=5.66, P<0.0001 | F(7,383)=3.12, P=0.0033 | Ethnicity: F(7, 383)=7.82, P<0.0001  Ethnicity*sex interaction:  F(15, 375)=5.72, P<0.0001 | | Ethnicity: F(7, 383)=2.50, P=0.0158  Ethnicity*sex interaction:  F(15,375)=2.80, P=0.0004 | |
| Notes: For individual parameter Wald test results: *P<0.05, **P<0.01, ***P<0.001. | | | | | | |

| **Table S5: Poisson regression results summary of association between ethnicity and mental health in MCS6 Outcome: SDQ internalising subscale; total sample n=10,357 (5298 girls; 5059 boys), weighted n=10,216)** | | | | | | |
| --- | --- | --- | --- | --- | --- | --- |
| **Ethnicity** | **Model 1: Unadjusted** | | **Model 2: Basic adjusted for household income** | | **Model 3: Overall adjusted model for all social support, participation, adversity factors and household income** | |
|  | **Girls**  **IRR (95% CI)** | **Boys IRR (95% CI)** | **Girls**  **IRR (95% CI)** | **Boys IRR (95% CI)** | **Girls**  **IRR (95% CI)** | **Boys IRR (95% CI)** |
| **White** n=8315 (4257 girls; 4058 boys) | 1.00 (ref) | 1.00 (ref) | 1.00 (ref) | 1.00 (ref) | 1.00 (ref) | 1.00 (ref) |
| **Mixed**  n=492 (246 girls; 246 boys) | 1.08 (0.94-1.26) | 0.93 (0.81-1.06) | 1.03 (0.89-1.19) | 0.90 (0.78-1.02) | 1.03 (0.90-1.17) | 0.94 (0.83-1.07) |
| **Indian**  n=275 (130 girls; 145 boys) | 0.74 (0.58-0.94)* | 0.98 (0.85-1.15) | 0.73 (0.57-0.93)* | 0.98 (0.86-1.12) | 0.78 (0.61-0.99)* | 1.07 (0.94-1.21) |
| **Pakistani**  n=496 (265 girls; 231 boys) | 1.19 (1.09-1.30)*** | 1.28 (1.12-1.47)*** | 0.94 (0.86-1.03) | 1.01 (0.88-1.17) | 0.97 (0.87-1.07) | 1.18 (1.01-1.38)* |
| **Bangladeshi**  n=221 (120 girls; 101 boys) | 1.09 (0.95-1.25) | 1.20 (0.95-1.52) | 0.85 (0.74-0.98)* | 0.95 (0.75-1.19) | 0.93 (0.81-1.07) | 1.10 (0.86-1.42) |
| **Black Caribbean**  n=102 (47 girls; 55 boys) | 1.02 (0.79-1.33) | 1.24 (0.99-1.57) | 0.89 (0.69-1.15) | 1.06 (0.85-1.32) | 0.96 (0.74-1.26) | 1.09 (0.92-1.30) |
| **Black African**  n=187 (93 girls; 94 boys) | 0.79 (0.66-0.94)** | 1.06 (0.82-1.36) | 0.67 (0.57-0.79)*** | 0.90 (0.72-1.12) | 0.74 (0.62-0.88)** | 1.03 (0.85-1.24) |
| **Other Ethnic Group**  n=269 (140 girls; 129 boys) | 1.15 (0.96-1.38) | 1.34 (1.08-1.65)** | 1.01 (0.85-1.20) | 1.23 (1.00-1.52) | 1.05 (0.90-1.23) | 1.30 (1.06-1.60)* |
| Adjusted Wald test results (association of ethnicity/outcome): | | | | | | |
|  | F(7, 383)=4.95, P<0.0001 | F(7,383)=2.95, P=0.005 | Ethnicity: F(7, 383)=4.34, P=0.0001  Ethnicity*sex interaction:  F(15, 375)=6.16, P<0.0001 | | Ethnicity: F(7, 383)=2.36, P=0.0227  Ethnicity*sex interaction:  F(15,375)=4.77, P<0.0001 | |
| Notes: For individual parameter Wald test results: *P<0.05, **P<0.01, ***P<0.001. | | | | | | |

| **Table S6: Poisson regression results summary of association between ethnicity and mental health in MCS6 Outcome: SDQ externalising subscale; total sample n=10,357 (5298 girls; 5059 boys), weighted n=10,216)** | | | | | | |
| --- | --- | --- | --- | --- | --- | --- |
| **Ethnicity** | **Model 1: Unadjusted** | | **Model 2: Basic adjusted for household income** | | **Model 3: Overall adjusted model for all social support, participation, adversity factors and household income** | |
|  | **Girls**  **IRR (95% CI)** | **Boys IRR (95% CI)** | **Girls**  **IRR (95% CI)** | **Boys IRR (95% CI)** | **Girls**  **IRR (95% CI)** | **Boys IRR (95% CI)** |
| **White** n=8315 (4257 girls; 4058 boys) | 1.00 (ref) | 1.00 (ref) | 1.00 (ref) | 1.00 (ref) | 1.00 (ref) | 1.00 (ref) |
| **Mixed**  n=492 (246 girls; 246 boys) | 1.06 (0.94-1.20) | 0.94 (0.84-1.06) | 1.00 (0.88-1.13) | 0.91 (0.81-1.02) | 1.02 (0.91-1.15) | 0.92 (0.82-1.03) |
| **Indian**  n=275 (130 girls; 145 boys) | 0.75 (0.60-0.92)** | 0.94 (0.82-1.08) | 0.73 (0.58-0.92)** | 0.93 (0.81-1.06) | 0.87 (0.70-1.08) | 1.03 (0.91-1.17) |
| **Pakistani**  n=496 (265 girls; 231 boys) | 1.14 (0.99-1.31) | 1.04 (0.94-1.15) | 0.88 (0.76-1.01) | 0.81 (0.73-0.90)*** | 1.05 (0.92-1.20) | 1.00 (0.90-1.12) |
| **Bangladeshi**  n=221 (120 girls; 101 boys) | 0.96 (0.84-1.08) | 0.95 (0.79-1.14) | 0.73 (0.65-0.83)*** | 0.74 (0.62-0.88)** | 0.91 (0.79-1.04) | 0.92 (0.77-1.10) |
| **Black Caribbean**  n=102 (47 girls; 55 boys) | 1.21 (0.97-1.51) | 1.29 (1.07-1.56)** | 1.03 (0.81-1.31) | 1.10 (0.89-1.35) | 1.11 (0.90-1.37) | 1.14 (0.98-1.34) |
| **Black African**  n=187 (93 girls; 94 boys) | 0.89 (0.79-1.01) | 0.88 (0.75-1.03) | 0.75 (0.67-0.85)*** | 0.74 (0.64-0.85)*** | 0.89 (0.78-1.02) | 0.87 (0.75-1.03) |
| **Other Ethnic Group**  n=269 (140 girls; 129 boys) | 1.11 (0.91-1.34) | 1.10 (0.94-1.28) | 0.96 (0.80-1.16) | 1.01 (0.86-1.18) | 1.06 (0.87-1.29) | 1.09 (0.94-1.27) |
| Adjusted Wald test results (association of ethnicity/outcome): | | | | | | |
|  | F(7, 383)=3.39, 0.0016 | F(7,383)=2.98, 0.0046 | Ethnicity: F(7, 383)=7.95, P<0.0001  Ethnicity*sex interaction:  F(15, 375)=16.76, P<0.0001 | | Ethnicity: F(7, 383)=1.44, P=0.1890  Ethnicity*sex interaction:  F(15,375)=10.51, P<0.0001 | |
| Notes: For individual parameter Wald test results: *P<0.05, **P<0.01, ***P<0.001. | | | | | | |
